# The differences in immunoadjuvant mechanisms of TLR3 and TLR4 agonists on the level of antigen-presenting cells during immunization with recombinant adenovirus vector

**DOI:** 10.1186/s12865-018-0264-x

**Published:** 2018-07-28

**Authors:** Ekaterina Lebedeva, Alexander Bagaev, Alexey Pichugin, Marina Chulkina, Andrei Lysenko, Irina Tutykhina, Maxim Shmarov, Denis Logunov, Boris Naroditsky, Ravshan Ataullakhanov

**Affiliations:** 1 National Research Center Institute of Immunology, Federal Medical-Biological Agency of Russia, Moscow, Russia; 2 Federal Research Centre of Epidemiology and Microbiology named after Honorary Academician N.F. Gamaleya, Ministry of Health, Moscow, Russia

**Keywords:** Toll-like receptors, Agonists, Adjuvants, Immunization, Recombinant non-replicating adenoviral vector, Expression of transgenes, Antigen-presenting cells, Dendritic cells

## Abstract

**Background:**

Agonists of TLR3 and TLR4 are effective immunoadjuvants for different types of vaccines. The mechanisms of their immunostimulatory action differ significantly; these differences are particularly critical for immunization with non-replicating adenovirus vectors (rAds) based vaccines. Unlike traditional vaccines, rAd based vaccines are not designed to capture vaccine antigens from the external environment by antigen presenting cells (APCs), but rather they are targeted to the de novo synthesis of vaccine antigens in APCs transfected with rAd. To date, there is no clear understanding about approaches to improve the efficacy of rAd vaccinations with immunoadjuvants. In this study, we investigated the immunoadjuvant effect of TLR3 and TLR4 agonists on the level of activation of APCs during vaccination with rAds.

**Results:**

We demonstrated that TLR3 and TLR4 agonists confer different effects on the molecular processes in APCs that determine the efficacy of antigen delivery and activation of antigen-specific CD4^+^ and CD8^+^ T cells. APCs activated with agonists of TLR4 were characterized by up-regulated production of target antigen mRNA and protein encoded in rAd, as well as enhanced expression of the co-activation receptors CD80, CD86 and CD40, and pro-inflammatory cytokines TNF-α, IL6 and IL12. These effects of TLR4 agonists have provided a significant increase in the number of antigen-specific CD4^+^ and CD8^+^ T cells. TLR3 agonist, on the contrary, inhibited transcription and synthesis of rAd-encoded antigens, but improved expression of CD40 and IFN-β in APCs. The cumulative effect of TLR3 agonist have resulted in only a slight improvement in the activation of antigen-specific T cells. Also, we demonstrated that IFN-β and TNF-α, secreted by APCs in response to TLR3 and TLR4 agonists, respectively, have an opposite effect on the transcription of the targeted gene encoded in rAd. Specifically, IFN-β inhibited, and TNF-α stimulated the expression of target vaccine antigens in APCs.

**Conclusions:**

Our data demonstrate that agonists of TLR4 but not TLR3 merit further study as adjuvants for development of vaccines based on recombinant adenoviral vectors.

**Electronic supplementary material:**

The online version of this article (10.1186/s12865-018-0264-x) contains supplementary material, which is available to authorized users.

## Background

Activation of antigen-specific T-cell responses is the basis of the most effective approaches in vaccination and immunotherapy [1–3]. Combination of vaccines with adjuvants significantly improves antigen-specific responses of T cells.

Agonists of Toll-like receptors 3 and 4 (TLR3 and TLR4) are effective adjuvants for various vaccines [4–7]. The mechanism of adjuvant action of TLR3 and TLR4 agonists is largely associated with their ability to activate APCs [8–13]. Particularly, TLR agonists enhance uptake and presentation of vaccine antigens by APCs (up-regulation of MHC class II and class I expression, inflammasome activation); induce maturation (increase expression of co-stimulating receptors and immunostimulatory cytokines), and migration of APCs to draining lymph nodes [8]. These events, induced in APCs by TLR agonists, determine the efficacy of antigen-specific CD8^+^ and CD4^+^ T cell responses after vaccination.

Vaccines based on recombinant adenoviral vectors (rAds) demonstrate high efficiency in activation of antigen-specific CD8^+^ and CD4^+^ T cells, which allows for the successful protection of animals against malaria, tuberculosis, Ebola, and other infectious diseases [14–17]. Theoretically, combination of adenoviruses with adjuvants could be a strategy to enhance the potential of rAd vaccines. However, there is no clear understanding of benefits resulting from the inclusion of adjuvants to the rAd vaccine composition. Despite the known examples of combinations of TLR3 and TLR4 agonists with rAds [18–24], the mechanisms of their adjuvant action is not well understood. It has been shown that signals from different TLRs are necessary to induce effective responses of antigen-specific T-cells upon immunization with rAd [25]. However, responses of CD8^+^ T cells in TLR-deficient mice (TLR2, 4, 5, 6, 7, 9) were not critically affected compared with wild-type mice. The effectiveness of vaccination was significantly decreased when the molecule MyD88 was knocked out. Therefore, an integrative action of individual TLRs in activation of MyD88 and induction of reliable responses to rAd immunization was proposed.

There is sufficient evidences that TLR3 and TLR4 agonists have an opposite effect on immunogenicity of rAd vaccines. Immunization with the rAd26-Gag vector in the presence of Poly I:C (TLR3 agonist) resulted in a decrease of Gag-specific responses of CD8^+^ T cells, whereas TLR4 agonists significantly improved ones [26]. Moreover, mice deficient in TLR3 and its adaptor molecule TRIF demonstrated an increase in antigen-specific T-cell responses compared with wild-type mice [25].

In contrast to protein and peptide vaccines that are designed to deliver vaccine antigens to APCs from outside, rAd-based vaccines require effective expression (de novo synthesis) of target antigens within APCs. Thus, the presentation of antigenic peptides in MHC-I and subsequent activation of CD8^+^ T cells is significantly dependent on the expression of adenoviral vector transgenes in APCs. Quinn et al. [27] had previously investigated the expression efficacy of target antigens in DCs isolated from lymph nodes of mice immunized with rAd. An opposing relationship between the expression of target antigens and the genes involved in interferon responses in APCs was observed. The external induction of the interferon genes by TLR3 agonist Poly I:C resulted in a reduction in both the total number of DCs in lymph nodes and the expression of target antigen of rAd. TBK1 (TANK-binding kinase 1) have been shown to be involved in the suppression of adenovirus vector expression through induction of an interferon response in splenocytes of immunized mice [28].

In a previous study, we have shown that the efficacy of rAd expression in APCs could be regulated with TLR agonists. In particular, we demonstrated that TLR4 agonists enhances the production of proteins encoded in rAd, while TLR3 agonist inhibits their production [29].

In the present study, we investigated the mechanisms of immunoadjuvant action of TLR3 and TLR4 agonists upon immunization with rAd (rAdTet-off H1) encoding haemagglutinin of influenza virus (H1). We demonstrated that TLR3 and TLR4 agonists equally enhance the generation of antigen-reactive CD4^+^ T cells in spleen of mice immunized with rAdTet-off H1. However, TLR3 and TLR4 agonists have shown different potential for activation of CD8^+^ T cell responses. The number of CD8^+^ T cells recognizing H1 was significantly improved in mice immunized with rAdTet-off H1 in combination with TLR4 compared to mice immunized with rAdTet-off H1 alone. TLR3 agonist did not improve responses of H1-reactive CD8^+^ T cells.

We carried out detailed experiments to study the efficiency of target adenovirus antigens presentation in DCs activated with TLR3 and TLR4 agonists. We investigated the effect of TLR3 and TLR4 agonists on the three types of processes in APCs that can provide the final adjuvant effect of each agonist: (1) the intensity of production of target H1 antigen; (2) the expression of co-activation receptors CD80, CD86 and CD40; and (3) the production of immunostimulatory cytokines.

## Results

### The immunoadjuvant effect of TLR3 and TLR4 agonists

**Fig. 1:**
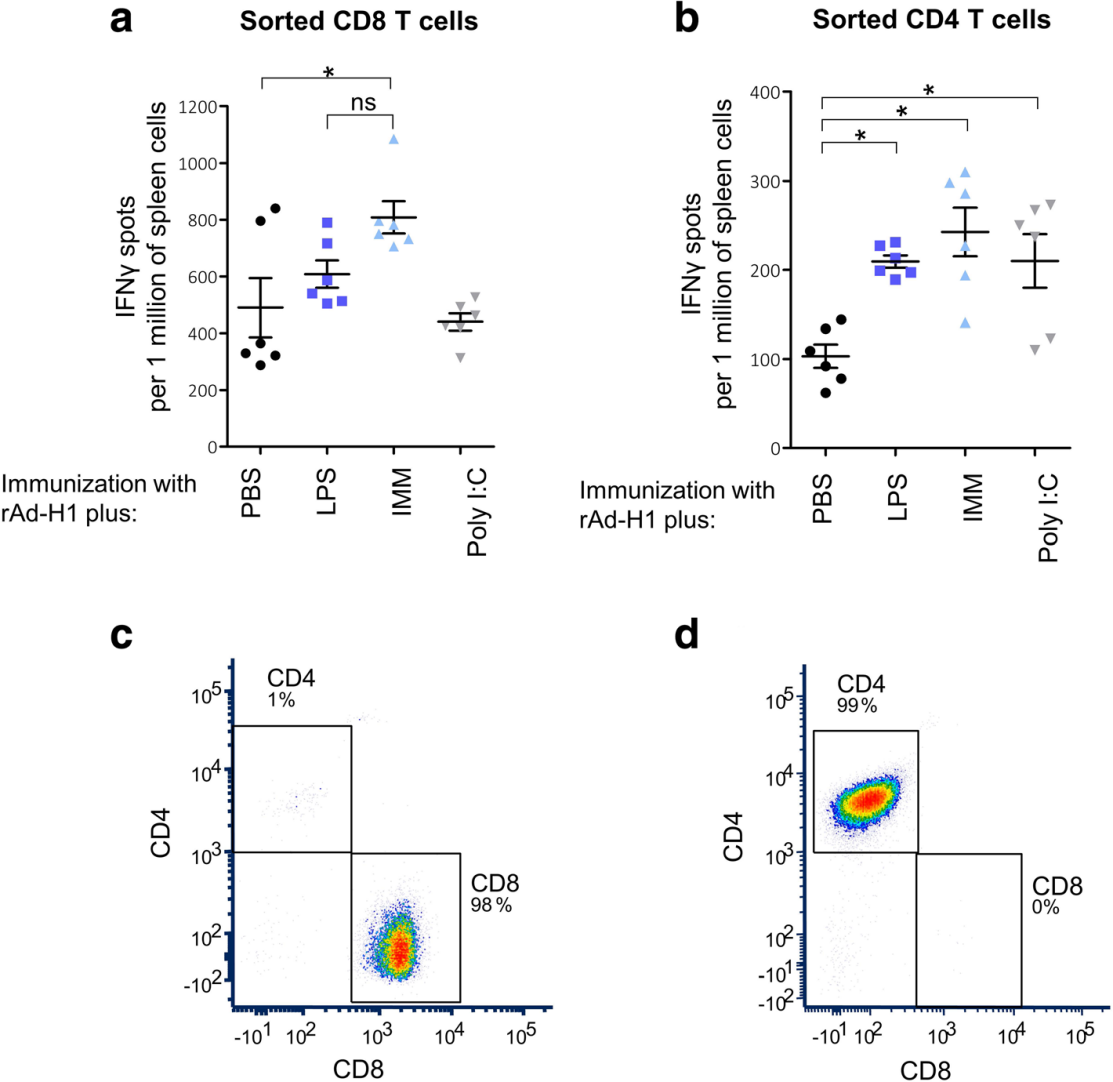
Immunoadjuvant effect of TLR3 and TLR4 agonists upon immunization with rAdTet-off H1 vaccine. Balb/c mice were immunized with 10^7^ PFU rAdTet-off H1 alone or in combination with 10 μg LPS, 10 μg IMM or 5 μg Poly I:C. Forty days after immunization mice were euthanized, sorted populations of CD8^+^ (**c**) and CD4^+^ (**d**) T cells from spleens of immune mice were co-cultured with bone marrow DCs loaded with rAdTet-off H1 (35 PFU/cell) (**a**) or with recombinant protein H1 (2 μg/ml) (**b**), respectively. Reactivated IFNγ-producing T cells were detected using ELISPOT method. The number of IFNγ-positive spots is shown as M + SD (per 1 million spleen cells). Significant differences (*p* < 0.05) between the control group of mice immunized with rAdTet-off H1 (PBS) alone and groups of mice immunized with rAdTet-off H1 in combination with TLR agonists (LPS, IMM or Poly I:C) are indicated by asterisks

BALB/c mice were vaccinated intramuscularly with rAdTet-off H1 (10^7^ PFU/mouse) alone (PBS) or in combination with TLR3 or TLR4 agonists. PBS or TLR agonists Poly I:C (5 μg), LPS (10 μg) or IMM (10 μg) were administered simultaneously (in one syringe) with rAdTet-off H1. Forty days after immunization mice were euthanized, and the number of antigen-reactive CD4^+^ and CD8^+^ T cells in the spleen of immunized mice were estimated with ELISPOT. The antigen-reactive populations of CD4^+^ and CD8^+^ cells were isolated from spleen of the mice by sorting on the FACS. Sorted CD4^+^ (Fig. 1d) and CD8^+^ (Fig. 1c) T cells were co-cultured with antigen loaded DCs.

The combination of rAdTet-off H1 with TLR4 agonist had significantly improved the magnitude of H1-specific T cell responses (Fig. 1). Up to 2–3 times more H1-specific CD8^+^ (Fig. 1a) and CD4^+^ (Fig. 1b) T cells had accumulated in the spleen of mice immunized with rAdTet-off H1 in combination with one of TLR4 agonists (LPS, IMM) compared to mice immunized with rAdTet-off H1 alone (PBS). TLR3 agonist induced an enhanced response of CD4^+^ T cells (Fig. 1b), however, it did not exert an adjuvant effect on H1-specific CD8^+^ T cells (Fig. 1a).

We concluded that TLR3 and TLR4 agonists enhance immune responses upon immunization with rAd based vaccines. An adjuvant effect of TLR agonists was primarily thought to affect APCs maturation which subsequently improves activation of antigen-specific T-cells [8–13]. Therefore, we further investigated the T-cells activation potential of APCs loaded with rAd and stimulated with agonists of TLR3 and TLR4.

### Activation of antigen-presenting dendritic cells with TLR3 and TLR4 agonists enhances the efficiency of responses of H1-specific T cells

Bone marrow derived dendritic cells (DCs) were activated with different concentrations of TLR3 or TLR4 agonists (0–10 μg/ml) and loaded with rAdTet-off H1 (3.5–350 PFU per cell) in vitro*.* Next, they were co-cultured with sorted CD4^+^ (Fig. 1d) and CD8^+^ (Fig. 1c) T cells from the spleen of the mice immunized with rAdTet-off H1. T cell responses were evaluated according to the number of T cells secreting interferon-γ (IFN- γ) using the ELISPOT.

**Fig. 2:**
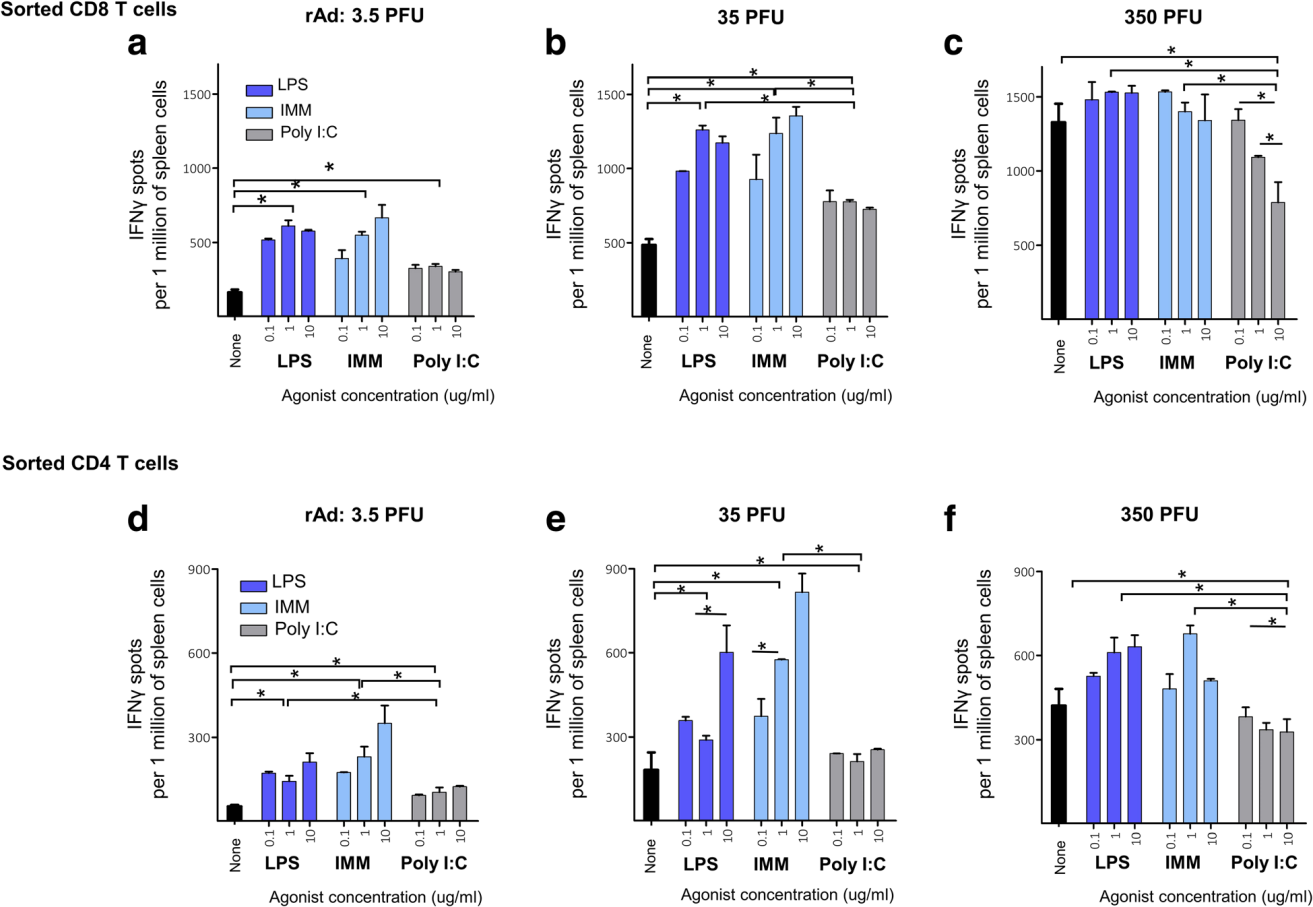
The effect of TLR3 and TLR4 agonists on the efficacy of reactivation of H1-specific T cells. **a-f** balb/c mice were immunized (i.m.) with 10^8^ PFU rAdTet-off H1. Forty days after immunization mice were euthanized, the pool of CD8^+^ (**a-c**) and CD4^+^ (**d**-**f**) T cells from the spleen of two immune mice was re-activated in vitro. Sorted CD8^+^ and CD4^+^ T cells were co-cultured with bone marrow derived DCs preloaded with 3.5 (**a**, **d**), 35 (**b**, **e**), or 350 (**c**, **f**) PFU/cell rAdTet-off H1 in the presence of 0–10 μg/ml agonists of TLR3 (Poly I:C) or TLR4 (LPS, IMM). The number of reactivated IFNγ-producing T-cells were detected by ELISPOT and calculated for 1 million spleen cells. Shown are M ± SD, statistically significant differences (*p* < 0.05) are indicated by asterisks

The number of IFN-γ-secreting T cells was dependent on the concentration of rAd loaded into the DCs (Fig. 2). As the viral dose increased from 3.5 (Fig. 2a, d) to 350 (Fig. 2c, f) PFU per cell, the number of IFN-γ-secreting CD8^+^ (Fig. 2a-c) and CD4^+^ T cells (Fig. 2d-f) increased from 100 (Fig. 2a) to 1500 (Fig. 2c), and from 80 (Fig. 2d) to 500 cells (Fig. 2f) (per 1 million spleen cells), respectively.

Activation of rAd-loaded DCs with TLR3 and TLR4 agonists resulted in an increase in the number of antigen-activated T cells. When APCs were loaded with a minimal viral dose, the minimal responses of antigen-reactive CD8 + T cells were observed (Fig. 2a). Activation of DC with TLR3 and TLR4 agonists allowed an increase of the minimal responses of antigen-reactive CD8^+^ T cells 4-fold (*p* = 0.0005) and 7-fold (*p* = 0.0008), respectively (Fig. 2a). At the medium rAd concentration only 1.5-fold (*p* = 0.0028) and 2.5-fold (*p* = 0.0004) improvement of responses of CD8^+^ T cells (Fig. 2b) by TLR3 and TLR4 agonists, respectively, were observed. At the maximum load of rAd, TLR3 agonist suppressed CD8^+^ T cell responses, while agonists of TLR4 did not affected responses of CD8^+^ T cells (Fig. 2c).

TLR3 or TLR4 agonists similarly affected CD4^+^ population of T cells. TLR3 agonist doubled CD4^+^ responses upon low virus load (Fig. 2d) and had no influence at the medium (Fig. 2e) or maximum (Fig. 2f) concentrations of rAd. TLR4 agonists increased the number of reactivated CD4^+^ T cells at low, medium and maximum concentrations of the rAd in 5, 3 and 1.5-times, respectively (Fig. 2d-f).

**Fig. 3:**
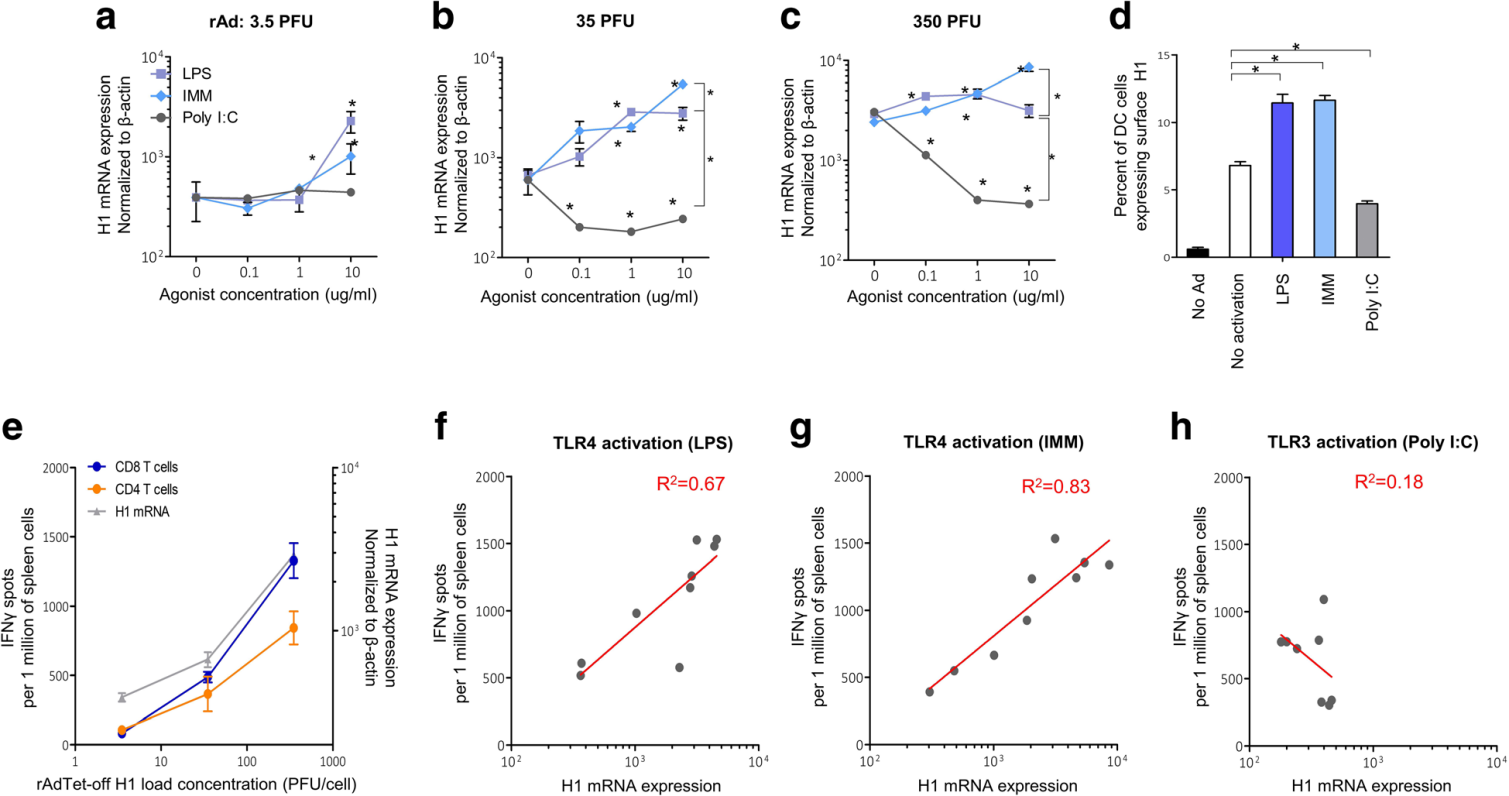
Efficacy of reactivation of antigen-specific T cells depending on the level of expression of the target rAd antigen in APCs. **a-c** the relative level of expression mRNA of H1 in DCs loaded with 3.5 (**a**), 35 (**b**), or 350 (**c**) PFU/cell rAdTet-off H1 in the presence of 0–10 μg/ml agonists of TLR3 (Poly I:C) or TLR4 (LPS, IMM). cDNA was obtained from total RNA extracts of DC and used as a template for quantitative PCR with primers specific for *H1* and *β-actin* genes. The expression values of *H1* gene were normalized with the expression of *β-actin* gene. **d** DCs were transduced with rAdTet-off H1 (100 PFU per cell) in the presence of 10 μg/ml agonists of TLR3 (Poly I:C) or TLR4 (LPS, IMM), 24 h after transfection cells were stained with primary (H1-specific) and secondary fluorochrome labeled antibodies, the percentage of H1-positive DCs in the test samples was detected by flow cytometry. Shown are M ± SD, statistically significant (p < 0.05) differences are indicated by asterisks. **e** dependence of H1-specific T cells reactivation efficiency and rAdTet-off H1 mRNA expression from the viral loading of DCs. **f-h** correlation of rAdTet-off H1 mRNA expression in DCs activated with TLR4 agonists – LPS (**f**), IMM (**g**), and TLR3 agonist Poly I:C (**h**) with an efficiency of reactivation of H1-specific T cells

T-cell responses were dependent on the expression level of the target H1 antigen in DCs (Fig. 3e). Stimulation of DCs with TLR4 agonists increased the production of the target antigen H1 (Fig. 3a-d, Additional file 1: Figure S1) in APCs. There was a direct association between the level of expression of H1 and the reactivation efficiency of CD4^+^ or CD8^+^ T cells (Fig. 3f, g) upon activation of DC with TLR4 agonists. Production of the H1 in DCs was suppressed when antigen-presenting DCs were exposed to TLR3 agonist (Fig. 3a-d, Additional file 1: Figure S1) and no correlations between the expression of the target antigen and the responses of the T cells were observed (Fig. 2h). TLR3 agonist stimulated T cell responses at medium rAd load (Fig. 2a, b, d, e), but TLR3 agonist inhibited T cell responses at a higher concentration of rAds (Fig. 2c, f). This denotes that the production of the target antigen in the DCs activated with TLR4 agonists positively contributes to the effectiveness of T cell activation, but this does not occur when the DCs are activated with TLR3 agonist.

TLR3 and TLR4 agonists influenced the activation of T cells, regardless of the type of APCs (DCs and macrophages) used to present the rAd antigens (Additional file 1: Figure S2).

### The stimulation of co-activation molecules and pro-inflammatory cytokines in antigen-presenting cells necessary for effective activation of antigen-reactive T cells

The effective stimulation of T cells in addition to the successful presentation of the target antigen in MHCI or MHCII complexes (signal 1 activating the T cell via TCR/CD3) requires at least two additional activation signals. The T cell receives the second signal through the CD28 and CD40L, which arises from binding the co-stimulating molecules CD80, CD86, CD40 on the surface of the APCs and ensures signals for T-cell stimulation and secretion of pro-inflammatory cytokines in APCs [30]. The source of the third signal are pro-inflammatory cytokines and type 1 interferons [31–36]. They maintain survival of antigen-specific T cells and development of productive antigen-specific reactions.

**Fig. 4:**
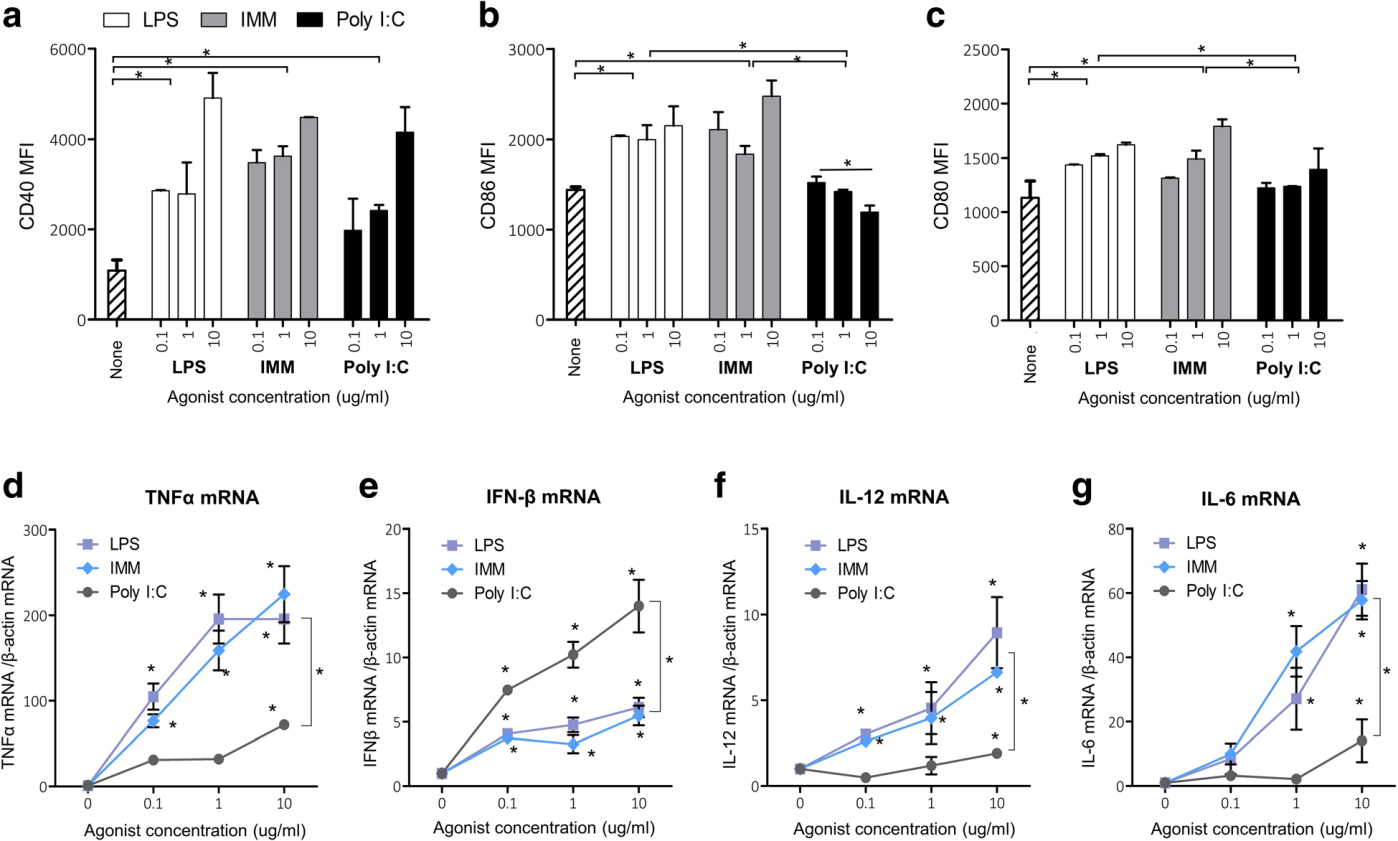
Expression of co-activation markers CD40, CD80 and CD86, proinflammatory cytokines TNFα, IL-12, IL-6 and interferon-β in DCs activated with TLR3 and TLR4 agonists. **a**-**c** DCs were transfected with rAd-GFP (100 PFU/cell) and cultivated for 24 h in the presence of 0–10 μg/ml TLR3 (Poly I:C) or TLR4 (LPS, IMM) agonists. Cells were stained with fluorochrom-labeled antibodies specific to CD40 (**a**), CD86 (**b**), CD80 (**c**) and the mean fluorescence of the samples was detected by flow cytometry. **d**-**g** DCs were incubated for 2 (**d**, **e**) and 7 (**f**, **g**) hours in the presence of 0–10 μg/ml agonists of TLR3 (Poly I:C) or TLR4 (LPS, IMM). cDNA was obtained from total RNA extracts of DC and used as a template for quantitative PCR with primers specific for *TNF-α* (**d**), *IFN-β* (**e**), *IL12* (**f**), and *IL6* (**g**) and *β-actin* genes. The expression values of cytokine’s genes were normalized with expression of *β-actin* gene. Values of mRNA expression after activation were normalized to the same values before activation (point 0). Shown are M ± SD, statistically significant (*p* < 0.05) differences are indicated by asterisks

We measured the expression of key co-stimulatory molecules (CD80, CD86 and CD40) and pro-inflammatory cytokines (IL12, TNF-α, IL6 and IFN-β) in DCs activated with TLR3 and TLR4 agonists (Fig. 4).

TLR4 agonists significantly enhanced the expression of co-activation receptors CD40, CD80 and CD86 on the surface of DCs (Fig. 4a-c). TLR3 agonist induced increased expression of CD40 (Fig. 4a) but had not affected expression of CD80 and CD86 (Fig. 4b, c).

DCs activated with TLR3 and TLR4 agonists also differed in the level of induced expression of the genes of pro-inflammatory cytokines. TLR4 agonists significantly stimulated transcription of *TNF-a*, *IL12* and *IL6* genes in DCs (Fig. 4d, f, g). TLR3 agonist activated *TNF-a*, *IL12* and *IL6* genes only at very low levels compared to the TLR4 agonist. However, TLR3 agonist stimulated production of IFN-β mRNA to a greater extent, than TLR4 agonists (Fig. 4e).

### Enhancement and suppression of the adenoviral vector expression in antigen-presenting cells by TLR4 and TLR3 agonists can be transmitted through the secretion of cytokines in a paracrine manner

In response to activation with TLR agonists, antigen-presenting cells (dendritic cells and macrophages) secrete pro-inflammatory cytokines, chemokines, interferons and other biologically active substances capable of paracrine influence on the neighboring cells. We demonstrated that TLR4 agonists preferentially stimulate the production of pro-inflammatory cytokines, such as TNF-α, IL-6, IL-12, but TLR3 agonist activated type 1 interferons (Fig. 4). We hypothesized that differences in the spectrum of activated cytokines could determine the different effects of TLR4 and TLR3 agonists on the expression of the adenovirus vector in antigen-presenting cells. To test this, we investigated the possibility of paracrine transfer of TLR-agonist induced regulation of the target protein expression from activated APCs to non-activated ones.

**Fig. 5:**
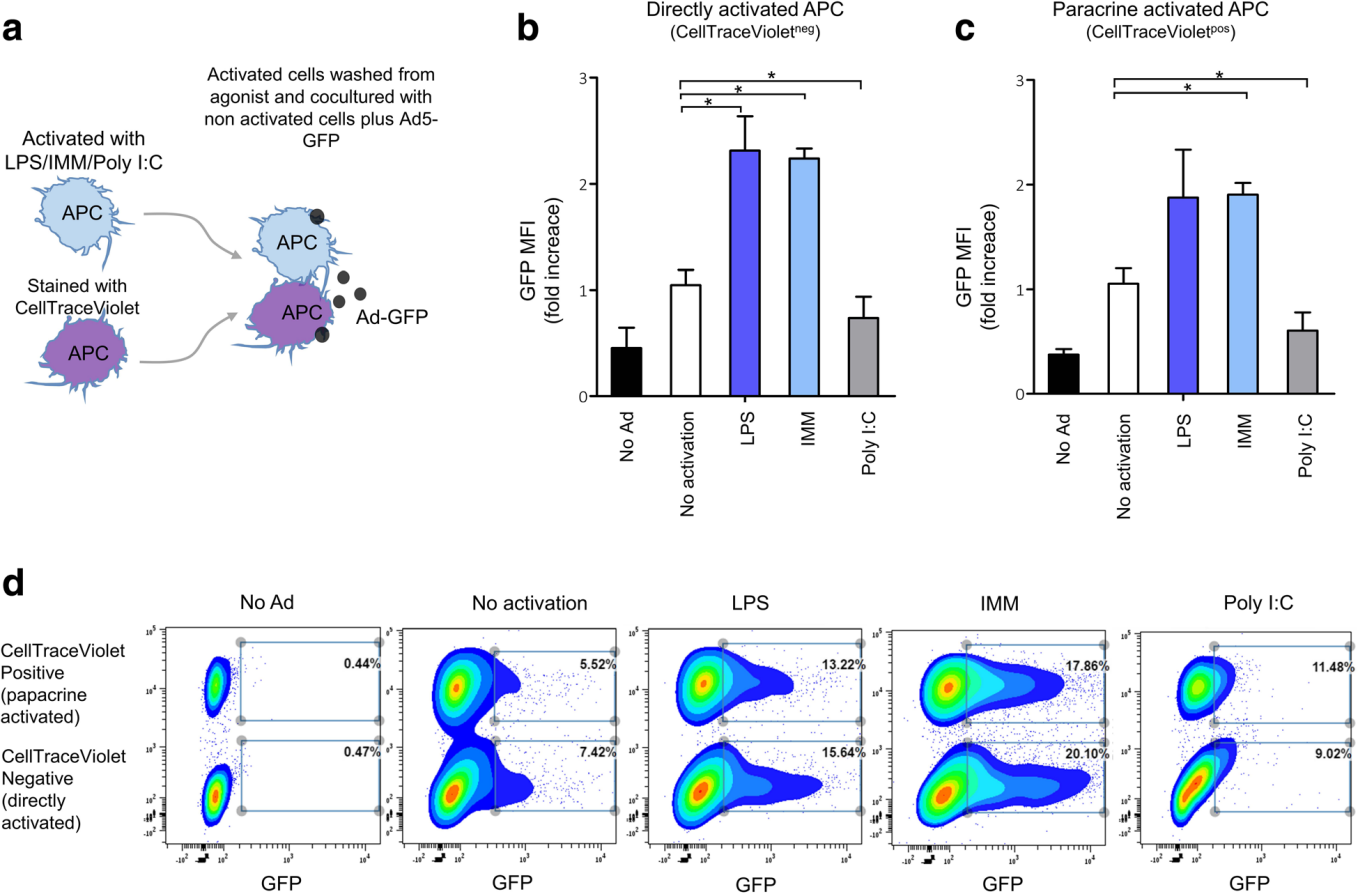
Paracrine transfer of enhancement and suppression of rAd expression in APCs. **a** Scheme of the experiment: peritoneal macrophages were incubated for 3 h in the presence of 10 μg/ml agonists of TLR3 (Poly I:C) and TLR4 (LPS, IMM) or without activators, cells were washed three times from the activators and mixed with non-activated Celltrace ™ Violet-labeled cells. Mixed populations of cells were transfected with rAd-GFP (100 PFU/cell), as a negative control - cells without transduction (no Ad). After 24 h, a mean fluorescence intensity of GFP (**b**, **c**) and percentage of GFP-positive cells (**d**) were measured in both populations of cell. Shown are M ± SD, statistically significant (*p* < 0.05) differences are indicated by asterisks

APCs were pre-activated with TLR agonists, washed from the agonists and cultured together with non-activated APCs. The combined culture of activated and non-activated APCs was transduced with rAd-GFP. Non-activated APCs were pre-labeled with the fluorescent dye Celltrace™ Violet, which further allowed us to distinguish two cell populations by flow cytometry. Intensity of production of the rAd-coded GFP protein was analysed in each of the two populations of cells (Fig. 5a). Enhanced expression of GFP was observed in APCs, which were pre-activated by TLR4 agonist. TLR4 agonist induced a 2-fold enhancement of the percentage and almost 2-fold increased mean fluorescence intensity of GFP-positive APCs (Fig. 5b, *p* = 0.011–0.041). Cells pre-activated with TLR3 had a lower GFP expression than cells transduced with rAd-GFP without TLR agonist activation. TLR3 agonist induced a decrease of mean fluorescence intensity of cells in the GFP channel (Fig. 5b, *p* = 0.015), while the percentage of cells producing GFP did not change significantly (Fig. 5d, *p* > 0.05).

Non-activated APCs following co-culturing with APCs pre-activated with TLR4 agonist expressed enhanced levels of GFP (Fig. 5c, *p* = 0.037) in the same manner to APCs directly activated with TLR4 agonist (Fig. 5b, *p* = 0.011–0.041). GFP synthesis was suppressed in non-activated APCs following co-culturing with APCs pre-activated with TLR3 agonist (Fig. 5c, *p* = 0.01), as well as in APCs directly activated by with TLR3 agonist (Fig. 5b, *p* = 0.015).

As can be seen from the data presented (Fig. 5), the effects of enhancement and suppression of GFP expression appeared both in cells directly activated with TLR4 or TLR3 agonists (Fig. 5b) and in non-activated cells neighboring with TLR3- or TLR4-activated APCs (Fig. 5c). Therefore, APCs activated with TLR4 or TLR3 agonists secrete signals that determine the enhancement or suppression of the production of the target rAd protein. Such signal molecules paracrinally affect non-activated APCs and provide an enhancement/suppression of rAd expression in APCs without TLR3 and TLR4 agonists’ activation.

### The nature of paracrine signals determining enhancement or suppression of rAd expression in antigen-presenting cells

As indicated above, the enhancement (TLR4 agonists) and suppression (TLR3 agonist) of rAd expression in APCs occurs at the transcription level of the target gene (Fig. 3a-c) and can be paracrinally transmitted (Fig. 5). We suggested that cytokines and interferons are capable of playing the role of mediators of paracrine action of TLR-activated APCs on non-activated APCs.

The rAds used in this study encoded target genes (H1 or GFP) under the control of the NF-kB-dependent CMV promoter. TLR4 agonists have stimulated production of TNF-α (Fig. 4d), which is an effective activator of NF-kB in APCs [37]. Therefore, we proposed TNF-α mediated activation of transcription of the target gene under the NF-kB-sensitive promoter providing an enhanced expression of the target gene of rAd in APCs. In our experiments, TLR3 agonist caused enhanced production of IFN-β (Fig. 4e) and inhibited the expression of the target rAd gene in the APCs (Fig. 3a-d). Type 1 interferons are well known antiviral effectors providing destruction of viral RNA [38]. We assumed that IFN-β, secreted by TLR3-stimulated APCs, could suppress the expression of the viral vector accelerating the degradation of mRNA of the target antigen in APCs. To prove our assumptions, we used recombinant TNF-α and IFN-β proteins.

**Fig. 6:**
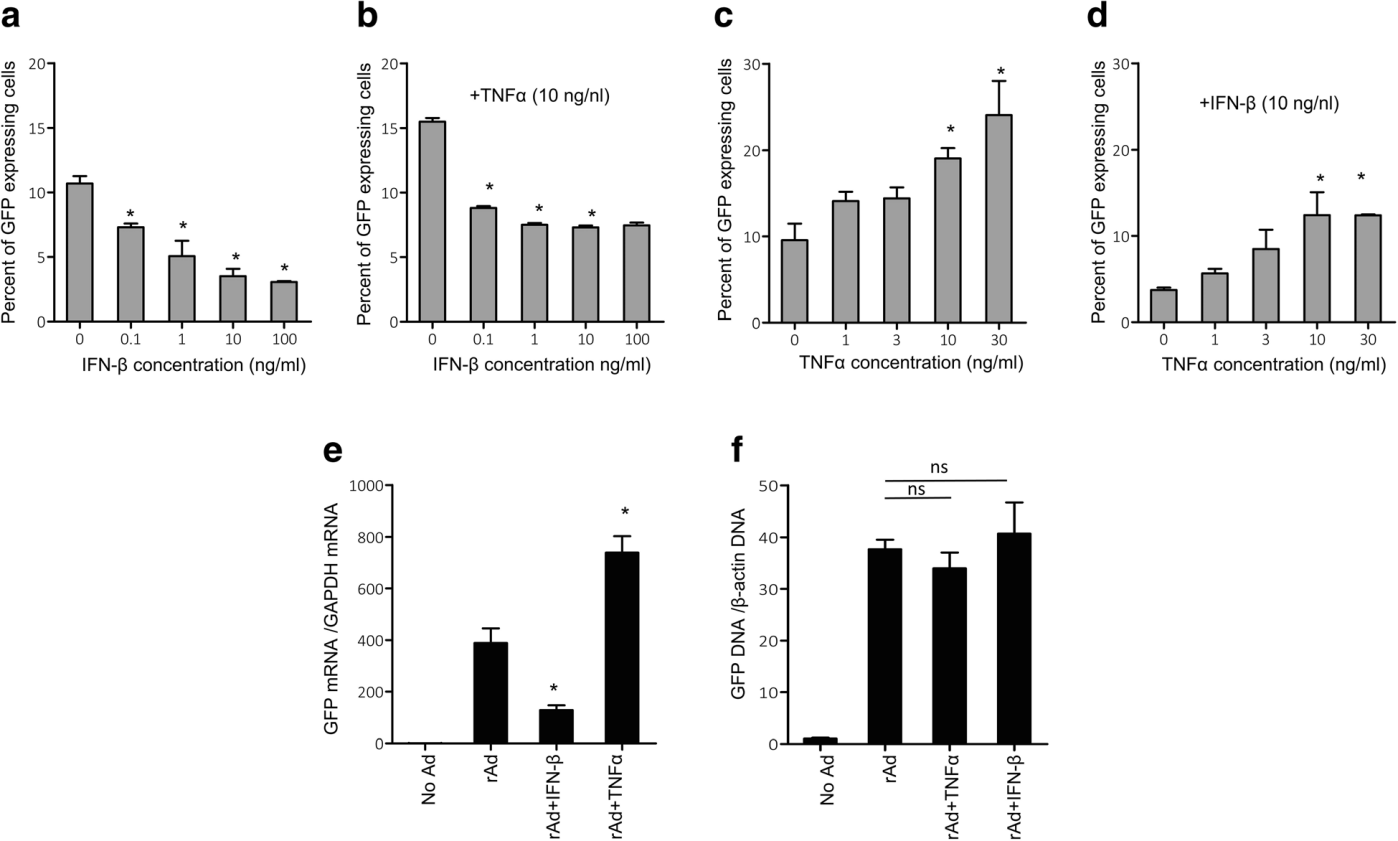
Effect of TNFα and IFNβ on the expression of rAd in APCs**.** Peritoneal macrophages were transfected with rAd-GFP in the presence of 0–30 ng/ml of TNF-α (**c**, **e, f**), 0–100 ng/ml IFN-β (**a**, **e, f**), or in the presence of combinations of 0–100 ng/ ml IFN-β and 10 ng/ml TNF-α (**b**), 0–30 ng/ml TNF-α and 10 ng/ml IFN-β (**d**). After 24 h, the efficacy of GFP protein synthesis (**a**-**d**), the level of transcription of the *GFP* (**e**) gene mRNA and the amount of viral DNA penetrated into the cells upon transfection (**f**) were analyzed. **a**-**d** GFP fluorescence in cells was detected by cytometry, mRNA (**e**) and DNA (**f**) from the lysates of DCs were extracted, RNA was subjected to a reverse transcription reaction; the obtained cDNA (**e**) and total cellular DNA (**f**) were used as a template for quantitative PCR with primers specific for *GFP*, *GAPDH* and *β-actin* genes. The expression values of *GFP* gene were normalized with the expression of *β–actin* or *GAPDH* genes. Values of GFP mRNA and DNA were normalized to the control values (No rAd). Shown are M ± SD, statistically significant (*p* < 0.05) differences are indicated by asterisks

Antigen-presenting cells were transfected with rAd-GFP in the presence of recombinant TNF-α (0–30 ng/ml) (Fig. 6c) or IFN-β (0–100 ng/ml) (Fig. 6a). IFN-β suppressed (Fig. 6a), and TNF-α enhanced (Fig. 6c) the production of GFP in APCs in a dose-dependent manner. The combined effect of cytokines TNF-α + IFN-β (Fig. 6b, d) resulted in a decrease in the effects of each cytokine alone (Fig. 6a, c). In particular, the enhanced expression of GFP induced by TNF-α decreased when IFN-β was added (Fig. 6d), and conversely, the inhibited expression of GFP in the presence of IFN-β enhanced following addition of TNF-α (Fig. 6b). The observed effects indicate that the regulation of mRNA transcription of rAd-GFP in APC could be mediated by IFN-β and TNF-α (Fig. 6e). TNF-α increases, and IFN-β inhibits (Fig. 6e) expression of target GFP mRNA in adenovirus infected APCs. The studied cytokines had no effect on the content of viral DNA in transfected cells (Fig. 6f) and, therefore, did not affect the efficiency of virus entry into APCs. Therefore, the observed effects were solely dependent on the transcription of the target antigens.

## Discussion

Non-replicating recombinant adenoviral vectors (rAd) are an effective platform for the development of vaccines against a wide range of pathogens and diseases [14–17]. Combinations of rAd vaccines with adjuvant substances are capable of increasing the intensity of responses of antigen specific CD4^+^ and CD8^+^ T cells and enhancing the protective effect followingimmunization. TLR agonists demonstrated a high adjuvant potential when administered together with various adenoviral vectors [7, 18, 19, 21–23, 26, 39–43]. At the same time, there was no sufficient information about the mechanisms of immunoadjuvant action of agonists of TLR receptors. Effects of TLR agonists on the processes of presentation of target antigens of rAd in APCs and on the processes of activation of antigen-reactive T cells were poorly understood as well.

In this work we studied the mechanisms of adjuvant action of TLR3 and TLR4 agonists combined with rAd vaccine. We used Poly I:C and LPS as a classical well-studied agonists of TLR3 and TLR4 receptors with high immunostimulatory activity [44]. IMM (Immunomax®) is a pharmaceutical grade plant derived injectable TLR4 ligand. It is a large water-soluble polysaccharide which structure is solved [45]. In vitro IMM directly activates DCs and macrophages [29, 46, 47]. Activated DCs express co-activation receptors for T cells (CD86, CD80 and CD40), and secrete immunostimulatory cytokines (IL-12, IL-6, TNF-α, RANTES, MIP-1, MCP-1, IL-1), and antimicrobial substances (type I interferons, NO, peroxynitrite, CRAMP). After activation with IMM both types of APCs, DCs and macrophages, acquire antitumor properties, although their cancer-killing mechanisms are different [46]. The influence of IMM on DCs and macrophages is equivalent to LPS [29, 46, 47]. Using InvivoGen HEK-Blue™ TLR cell lines which express certain human TLRs, it has been shown that IMM activates only cells which express TLR4 but not those which express TLR2, TLR3, TLR5, TLR7, TLR8 or TLR9 [47]. IMM induces activation of MyD88 → NF-*k*B signaling axis. DCs’ activation with IMM is abrogated with CLI-095, a specific inhibitor of TLR4 intracellular domain [29]. It also activates mouse and human NK cells and this activation is DC-mediated [47]. IMM does not activate DCs/macrophages obtained from the TLR4−/−knockout mice [46]. In vivo injections of IMM substantially changed cellular composition of spleen and lung of mice having 4 T1 breast cancer metastatic disease. It increased frequency of activated NK, CD4^+^ and CD8^+^ cells, and decreased frequency of myeloid-derived suppressor cells in spleens of mice [47]. In primary 4 T1 tumor-resected mice, repeated injections of IMM significantly prolonged overall survival and cured 30% of animals [47].

Administration of TLR3 and TLR4 agonists together with rAdTet-off H1 vaccine vector encoding haemagglutinin from the influenza virus (H1) significantly increased the intensity of responses of T-cells recognizing H1 epitopes (Fig. 1). Agonists of TLR3 (Poly-I:C) and TLR4 (LPS, IMM) doubled the amount of antigen-reactive CD4^+^ T cells secreting IFN-γ in the spleen of mice immunized with rAdTet-off H1 (Fig. 1b). TLR4 agonists also increased the accumulation of antigen-reactive CD8^+^ cells (Fig. 1a), while TLR3 agonist did not influence the responses of antigen-reactive CD8^+^ T cells (Fig. 1a). Cytotoxic CD8^+^ T-cells have a key role in the elimination of intracellular infectious agents, at the same time, CD4^+^ T helper cells are able to enhance the effector function of CD8^+^ T cells [48, 49]. Thus, the ability of TLR4 agonists to improve both types of T-cell responses - CD4^+^ and CD8^+^ − is an important adjuvant property.

The adjuvant effect of TLR agonist is primarily associated with the activation of APCs [8–13]. TLR agonists up-regulate expression of co-stimulatory and MHC molecules, increasing antigen presentation properties, induce synthesis of cytokines and chemokines, facilitating the formation of a more intense immune response against foreign antigens [8]. We carried out detailed study of the effects of TLR3 and TLR4 agonists on the presentation of rAd antigens in DCs using in vitro models where DCs transduced with rAdTet-off H1 was stimulated with TLR3 or TLR4 agonists and co-cultured with H1-reactive T cells.

TLR3 and TLR4 agonists differently reactivated T cells specific to rAdTet-off H1 (Fig. 2, Additional file 1: Figure S2). TLR4 agonists increased the number of reactivated CD4^+^ and CD8^+^ T-cell at 4–10 times more. This allows a 10-fold decrease in the dose of the virus vector inducing a maximum response of T cells. TLR3 agonist Poly I:C doubled the amount of reactivated CD8^+^ T cells co-cultured with DCs loaded with suboptimal doses of rAdTet-off H1. However, Poly I:C inhibited the responses of T cells reactivated with DCs loaded with maximum dose of rAd. Therefore, the adenovirus load of DC determines efficiency of antigen expression as well as reactivation of antigen-specific T cells. It can be proposed that studies demonstrating a positive effect of TLR3 agonist on rAd-immunisation was considered with non-maximal doses of rAd [7, 21, 22, 41, 42].

Activation of T cells following their interaction with complexes of antigens with MHCI/II depends on three key regulatory signals. The first signal occurs when the T cell receptor recognizes MHCI/II-antigen complex, which directly depends on the antigen binding affinity, and on the efficiency of target antigen expression in APCs in the case of adenoviral immunization. The second signal occurs, when T-cell receptors are linked to co-activation molecules (CD40, CD80, CD86) on the surface of APCs, which depends on it’s the expression on the surface of the DCs. Finally, the third signal is determined by the T-cell’s cytokine environment. In the absence of pro-inflammatory cytokines and type I interferons, activated T cells die before they have completed their function [31–36].

We investigated the influence of TLR3 and TLR4 agonists on each of three mentioned signals in antigen-presenting DCs. TLR4 agonists significantly increased expression of the target antigen encoded in rAd (Fig. 3a-d), stimulated expression of CD40, CD80 and CD86 molecules and enhanced secretion of pro-inflammatory cytokines TNFα, IL-12, IL-6, but not type I interferons (Fig. 4). TLR3 agonist suppressed expression of the target antigen encoded in rAd (Fig. 3b-d), and strongly activated expression of CD40, but not CD80 and CD86 molecules, and did not substantially stimulated expression of TNF-α, IL-12, IL-6 genes (Fig. 4). Simultaneously, DCs stimulated with TLR3 agonist (Poly I:C) produced a large amount of *IFN-β* (Fig. 4e). It is known that type I interferons determine maturation of DCs and regulation of T cell responses in the presence of a TLR3 agonist. Blocking interferon signaling in IFNabR^−/−^ mice resulted in a critical decrease of the CD40, CD86 proteins expression on the surface of DCs in the presence of Poly I:C and inhibition of antigen-specific T cell responses [50].

Cytokines and type I interferons produced by APCs in response to LPS, IMM and Poly I:C are able to determine the function of the third activation signal for T cells as well as the efficiency of production of the target rAd antigen in APCs. Both inhibition and enhancement of rAd expression could be regulated by cytokine signals. These signals are transmitted from the cells activated with TLR3 and TLR4 agonists to non-activated cells in a paracrine manner (Fig. 5). This is particularly shown for TNF-α and IFN-β, which are capable of enhancing and inhibiting the expression of the target antigen in APC transduced by rAd, respectively (Fig. 6). We demonstrated that intensive secretion of IFN-β promotes a decrease in expression level of rAdTet-off H1 in DCs stimulated with Poly I:C (Figs. 3b-d and 4e). In contrast, enhanced production of TNF-α following activation of APCs with LPS or IMM (Fig. 4d) promoted increased expression of rAd antigens in APCs (Fig. 3a-d). Previously, it was shown that the efficiency of rAd expression in vivo is determined by the interferon response of target cells [27, 28]. The expression of rAd in DCs and the induction of CD8^+^ T cells responses were inversely correlated with the expression level of genes responsible for activation of interferon pathways in APCs [27]. Interferon-β has a mixed effect on the activation of T cells upon their contact with APCs [27, 28, 33, 34]. Particularly, IFN-β had an antiviral effect and inhibited production of target antigen encoded in rAd in APCs [27, 28]. Stimulation of APCs with Poly I:C (Fig. 3b-d) and IFN-β (Fig. 6a) caused a suppressed antigen expression of rAdTet-off H1 in macrophages and DC, which resulted in a decreased reactivation of H1-specific T cells (Fig. 2).

**Fig. 7:**
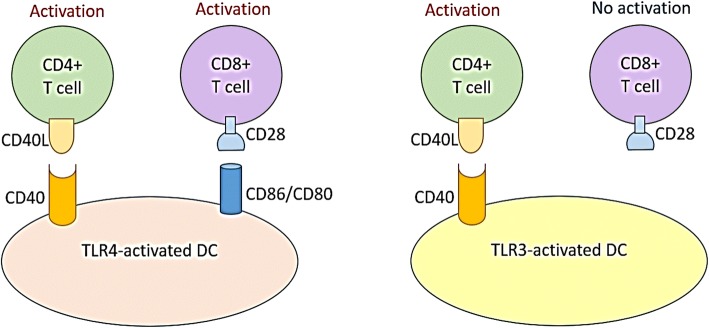
A schematic presentation of the molecular mechanisms responsible for different activating effects of TLR3- and TLR4-induced DCs on CD4+ and CD8+ T cell subsets

According to our data TLR3-activated DCs do express CD40 (Fig. 4a) but do not express CD86/80 (Fig. 4b and c). The expression of CD40 is a key co-stimulatory molecule for CD4^+^ T cells, because the CD40L is expressed preferentially by CD4^+^ T cells, but not CD8+ T cells [51]. Which means that TLR3-induced expression of CD40 on APCs should provide the co-stimulatory signal to CD4^+^ T cells but not to CD8^+^ T cells (Fig. 7). The latter are readily accepting the co-stimulatory signal generated via CD80/86-CD28 axis which is well induced by TLR4 but not TLR3 agonists.

## Conclusions

We have identified the principal differences in the adjuvant action of TLR3 and TLR4 agonists used for rAd based immunization. TLR3 agonist stimulates accumulation of antigen-specific CD4^+^ T cells, and TLR4 agonist increases accumulation of both CD4^+^ and CD8^+^ T cells in the spleen of rAd-immunized mice. The mechanisms of adjuvant action of TLR3 and TLR4 agonists were studied in the model of reactivation of antigen-specific T cells in vitro. Adjuvant action of TLR4 agonists accumulates from the stimulation of rAd target antigen expression, up-regulation of co-activation molecules and production of pro-inflammatory cytokines by antigen-presenting cells. These changes have a complex positive effect on the induction of T-cell responses to the target antigen. Poly I:C is not as strong as TLR4 agonists in stimulating the expression of co-activation molecules and cytokines. It even suppressed the rAd target antigen expression in APCs at high adenovirus load. The adjuvant effect of Poly I:C could be mediated by an elevated level of IFN-β and CD40 molecules.

## Methods

### Antibodies and reagents

Agonist of Toll-like receptor 4 lipopolysaccharide from *E. coli* serotype 055: B5 (LPS, Sigma, L-2880), agonist of Toll-like receptor 4 Immunomax® (IMM, Immapharma), agonist of Toll-like receptor 3, an synthetic analog of double-stranded RNA, Poly Inosine: Poly Cytidine acid (Poly I:С, Invivogen), recombinant tumor necrosis factor α (TNF-α, Sigma, T7539), recombinant interferon-β1 (IFN-β, BioLegend, 581,302), Influenza H1 (A/California/04/2009) Hemagglutinin/HA Antibodies (Sino biological, 11,055-RM10), recombinant hemagglutinin of influenza virus H1 (Sino biological, 11,085-V084), dihydrochloride DAPI (Sigma), CellTrace™ Violet (Invitrigen, C34557) were used in this study.

### Animals

Female 8*–*10 weeks old BALB*/*c mice were obtained from the Stolbovaya Nursery of the Russian Academy of Medical Science and fed standard rodent food under standard animal house conditions in the vivarium of the National Research Center Institute of Immunology FMBA of Russia. All handling and experimental procedures with animals were carried out in strict accordance with the rules of research work with laboratory animals of the Institute of Immunology of FMBA Russia (Order of November 12, 2015), certified by the Local Ethics Committee (Resolution 4/17 of July 13, 2017).

Forimmunization animals were treated with low (10^7^ PFU/mouse) and high (10^8^ PFU/mouse) doses of rAdTet-off H1. The low vaccine dose was administered alone or in combination with 10 μg LPS or 10 μg IMM or 5 μg Poly I:C.

### Antigen-presenting cells

All cell cultures were incubated in a complete medium (CM) based on DMEM with 25 mM HEPES supplemented with a cocktail of nonessential amino acids, 10% fetal bovine serum (FBS, HyClone, Cat # SV30160.03 endotoxin level ≤ 10 EU/ml), 2 mM L- glutamine, 1 mM sodium pyruvate, and 10 μg/ml gentamycin at 37^о^С in a 5% СO_2_ humidified atmosphere (all culture supplements were obtained from PanEco, Russia).

Bone marrow derived dendritic cells (DCs) were obtained in vitro by culturing bone marrow cells of BALB/c mice with a granulocyte-macrophage colony-stimulating factor (GM-CSF, BioLegend). Mice were euthanized in a CO2 chamber, and bone marrow was washed out from the femurs and the tibias, erythrocytes removed by osmotic shock, nuclear cells washed twice with PBS (Amresco, E404), followed by cultivation in a complete medium supplemented with 10 ng/ml GM-CSF (Sigma) for 7 days, media was changed at day 4. After 7 days of culture the non-adherent (dendritic cells) cells were gathered*.* Viability and purity were assayed with flow cytometry. Among viable cell (> 80% by DAPI staining) non-adherent population of cells were identified as 70% CD11b^+^CD11c^+^ dendritic cells (DCs).

Peritoneal macrophages were obtained by washing of the peritoneal cavity of euthanized BALB/c mice. The cells were pelleted by centrifugation, resuspended in CM, and cultured for 18–20 h at 37 °C in a humidified atmosphere of 5% СO2. Then non-adhesive cells were gently washed away with PBS. The remaining adherent cells comprised over 90% of macrophages assayed by flow cytometry analysis with F4/80 staining.

### Constructing of the recombinant replication-defective adenovirus vector with a gene insert

In the study, we used GMP-grade rAds, which were produced in Federal Research Centre of Epidemiology and Microbiology named after N.F. Gamaleya.

Plasmid pShuttle-tet-off-tTA, carrying regulatory elements of the tet-off system, was obtained by cloning the required areas from plasmids pTet-off and pTRE-Tight (Tet-Off & Tet-On Gene Expression System, Clontech, USA) into plasmid pShuttle-CMV, using KpnI and EcoRV restriction endonucleases. Hemagglutinin H1 codon-optimized nucleotide sequence (synthesized by Evrogen JSC, Moscow, Russia) were cloned into the pShuttle-Tet-off-tTA plasmid. As a result, we got plasmid pShuttle-Tet-off-H1opt.

Nucleotide sequence of GFP gene was cloned into pShuttle CMV vector obtained from “The Ad-Easy Adenoviral Vector System (Stratagene, 240009)” according to manufacture’s instruction. Also we used plasmid vectors pGreen (Carolina Biological Supply Company), p310D (pRcCMV-SEAP).

The Ad-Easy Adenoviral Vector System (Stratagene, 240,009) was used to construct rAd-GFP and rAd5-tet-H1opt, according to the manufacturer’s instructions.

The GFP and H1 inserts in the corresponding constructs pShuttle-CMV-GFP, and pShuttle-Tet-off-H1 were verified by restriction analysis using EcoRI, NotI and EcoRV endonucleases and PCR. The presence of the genes *gfp* и *h1* in rAd was confirmed by PCR. rAds were grown in HEK-293 cells and chromatographically purified. The effective titer of the rAd-GFP and rAdTet-off H1 preparations was estimated using the plaque-forming assay in the HEK-293 cell culture.

The negative endotoxin content of rAds was demonstrated in LAL-test, InvivoGen HEK-Blue™ TLR4 reporter cell line assay and in the rabbit pyrogen test.

### Transduction of cells with recombinant replication-defective adenovirus vectors

The cell cultures were transduced with rAd-GFP or rAdTet-off H1 at the dose of 3,5–350 PFU/cell in 150 μl of CM. The transduced cells were cultured in the presence or absence of LPS (0.1–10 μg/ml), Immunomax (1–10 μg/ml), Poly I:C (0.1–10 мкг/мл), TNF-α (1–30 ng/ml) or IFN-β (0.1–100 ng/ml).

### CD4^+^ и CD8^+^ T-cell immune responses to H1 upon rAdTet-off H1immunization

Quantity and phenotype of hemagglutinin H1 specific T-cell were detected using the ELISPOT technique.

Immunised mice were euthanized in a CO2 chamber, suspension of cells from spleen of mice was separated at ficoll gradient (density 1.09 g/cm3) and mononuclear cells were gathered. Then mononuclear cells were treated with antibodies to CD4 (BioLegend, 100,412) and CD8 (BioLegend, 100,752). To obtain pure population of CD4+ and CD8+ T-cells we used flow cytometer FACSAria II. We excluded doubled events by FCS-A and FCS-H parameters. Dead cells were excluded by DAPI staining. Purity of sorted populations was approximately 90–95%. The purified populations of CD4^+^ and CD8^+^ T cells from the spleen ofimmunized mice were reactivated in vitro by culturing with antigen-presenting dendritic cells.

For restimulation of CD4+ T cells in vitro, we used dendritic cells that represent antigenic epitopes of the H1 in the context of MHC class II. For this purpose, dendritic cells were further activated with lipopolysaccharide from *E. coli* (1 μg/ml) and loaded with recombinant H1 protein (2 μg/ml). We also stimulated CD4^+^ T cells with dendritic cells transduced with rAdTet-off H1 (3.5–350 pfu per cell) to detect cross-presentation events. To restimulate CD8^+^ T cells in vitro, dendritic cells in which antigenic epitopes of the rAdTet-off H1 are present in the context of MHC class I were used. In this case, the dendritic cells were transduced with rAdTet-off H1 (3.5–350 PFU per cell).

### ELISPOT

96-well plate for ELISPOT (mouse IFN-γ ELISPOT Kit, cat. 552,569, BD Biosciences) was loaded by 10,000 dendritic cells with 150 μl of complete culture medium per well. Antigen-presenting dendritic cells were transduced with rAdTet-off H1 (3.5–350 PFU per cell) or loaded with recombinant H1 protein (2 μg) and incubated overnight in the presence of 0.1–10 μg/ml of agonist of TLR3 or TLR4. On next day 50 μl of spleens cells suspension of interest − 100,000 CD4^+^ T-cells or 50,000 CD8^+^ T-cells, were added to each well. The plate was incubated for 23 h in CO_2_-incubator strictly in the horizontal avoiding shaking. Then the plate was treated with accordance to manufacturer protocol (BD Biosciences). Wells of the plate were several times washed with distilled water and then 3 times with washer buffer (BD Biosciences). We added to each well 100 μl of detecting antibodies to IFN-y and incubated them within 2 h at room temperature. Then we scoured them 4 times with washer buffer and added 100 μl/well streptavidin-peroxidase conjugate (BD Biosciences), incubating within 1 h. After the incubation we elaborately scoured wells with washer buffer and then by PBS solution. Quickly added chromogenic substrate of 3-amino-9-ethylcarbazole (BD Biosciences), and incubated them within 15 min, after that elaborately washed them with distilled water, and left wells to dry at room temperature.

We took photo of each well using binocular microscope MBS-10 (magnification × 4) and digital camera Levenhuk DCM800 with 1280 × 960 pxls resolution. Quantification of spots with IFN-y that had been formed by single cells in the wells of plate was done by using the software package ImageJ (National Institutes of Health, USA).

### Measurement of production intensity of the GFP and H1 proteins encoded by rAd

Intracellular GFP accumulation was estimated by flow cytometry on FACS Aria II (BD Biosciences). Fluorescence was excited with the 488 nm laser, and emission intensity was measured between 515 and 545 nm.

Expression of membrane-bound H1 was examined by staining of cells with HA-specific primary and fluorochrome labeled secondary (Invitrogen, A-11034) antibodies, followed by flow cytometry by FACS Aria II.

### Real-time PCR

Total RNA, DNA, and cDNA preparations were obtained using Sintol reagent kits (RNA-extran, S-sorb, RT reagent kit, respectively), following the manufacturer’s instructions. The samples of purified total RNA were additionally treated with DNAse (Thermo Scientific, USA). RT-PCR was carried out by using primers which are shown in Table 1 following cycling on the DTprime-4 amplifier (DNA Technology Inc., Russia): 94 °C (5 min), 40 cycles at 94 °C (10 s) and 62 °C (20 s) and elongation at 72 °C for 5 s. The progress of cycling was monitored using FAM fluorescence. Expression of target mRNA was normalized to expression of β-actin or GAPDH mRNA using 2^ΔΔCp method.

**Table 1:** Nucleotide sequences of the primers used in the work

№	Gene Name	Primer name	5′-- > 3′ sequence
1	*gfp*	EGFP-F	GACCACTACCAGCAGAACAC
		EGFP-R	CTTGTACAGCTCGTCCATGC
		EGFP-TP	FAM-AGCACCCAGTCCGCCCTGAGCA-RTQ
2	*b-actin*	β-actin-F	AGAGGGAAATCGTGCGTGAC
		β -actin-R	CAATAGTGATGACCTGGCCGT
		β -actin-TP	FAM-CACTGCCGCATCCTCTTCCTCCC-RTQ
3	*gapdh*	GAPDH-F	TTCACCACCATGGAGAAGGC
		GAPDH-R	GGCATGGACTGTGGTCATGA
		GAPDH-TP	FAM-TGCATCCTGCACCACCAACTGCTTAG-RTQ
4	*tnfa*	TNF-F	CATCTTCTCAAAATTCGAGTGACAA
		TNF-RV	TGGGAGTAGACAAGGTACAACCC
		TNF-TP	FAM-CACGTCGTAGCAAACCACCAAGTGGA-RTQ
5	*H1*	H1-F	CTGGATGGATTCTGGGCAAC
		H1-R	CAGGGTAGCATGTGCCGTTG
		H1-TP	FAM –TGTGAATCCCTGAGCACCGCCT-RTQ
6	*ifnb*	IFN-F	ACCACAGCCCTCTCCATC
		IFN-RV	GCATCTTCTCCGTCATCTCC
		IFN-TP	FAM-CAACCTCACCTACAGGGCGGAC-RTQ

### Statistical analysis

Data are reported as means (M) ± standard deviation (SD). Statistical significance between groups was determined using the one-way ANOVA with Tukey post hoc comparison test or two-tailed unpaired t-test (*p* values < 0.05 were considered significant). All statistical parameters were calculated using GraphPad prism 5.0 Software.

## Additional file

Additional file 1: **Figure S1. **Effect of TLR agonists on the expression of rAd in DCs. DCs were transduced with rAdTet-off H1 (100 PFU per cell) in the presence of 10 μg/ml agonists of TLR3 (Poly I:C) or TLR4 (LPS, IMM); 24 h after transfection cells were stained with primary (H1-specific) and secondary fluorochrome labeled antibodies. The mean fluorescence (MFI) of H1-positive DCs in the test samples was detected by flow cytometry. Shown are M ± SD, statistically significant (*p* < 0.05) differences are indicated by asterisks. **Figure S2. **Effect of TLR3- and TLR4-activated APCs on the reactivation of CD4^+^ and CD8^+^ T-cells. Balb/c mice were immunized (i.m.) with 10^8^ PFU rAdTet-off H1. Forty days after immunization, the pool of CD8^+^ (**a, c**) and CD4^+^ (**b**, **d**) T cells from the spleen of euthanized immune mice was re-activated in vitro. Sorted CD8^+^ and CD4^+^ T cells were co-cultured with bone marrow derived DCs (**c**, **d**) or macrophages (MF) (**a**, **b**) preloaded with 20 PFU/cell rAdTet-off H1 in the presence of 0–10 μg/ml agonists of TLR3 (Poly I:C) or TLR4 (LPS, IMM). The number of reactivated IFNγ-producing T-cells were detected by ELISPOT and calculated for 1 million spleen cells. Shown are M ± SD, statistically significant differences (*p* < 0.05) are indicated by asterisks. (PDF 123 kb)

## Abbreviations

APCs: Antigen-presenting cells
BSA: Bovine serum albumin
DAPI: 4,6-diamidino-2-phenylindole
DCs: Dendritic cells
GAPDH: Glyceraldehyde 3-phosphate dehydrogenase
GFP: Green fluorescent protein
GM-CSF: Granulocyte-macrophage colony-stimulating factor
H1: Haemagglutinin of a human influenza virus H1 subtype
i.m.: Intramuscular injection
IMM: Immunomax
LPS: Lipopolysaccharide
M: Mean
MHC: Major histocompatibility complex
PBS: Phosphate*-*buffered saline
PFU: Plaque-forming unit
Poly I:C: Polyinosinic-polycytidylic acid
rAD: Recombinant adenovirus vector
SD: Standard deviation
TLR: Toll-like receptors
