# Supplementary material for: The differences in immunoadjuvant mechanisms of TLR3 and TLR4 agonists on the level of antigen-presenting cells during immunization with recombinant adenovirus vector
Source: BMC Immunol. 2018 Jul 28;19:26. doi: 10.1186/s12865-018-0264-x (PMC6064145; doi:10.1186/s12865-018-0264-x)
Supplement: Supplementary file 1 — Figure S1. Effect of TLR agonists on the expression of rAd in DCs. DCs were transduced with rAdTet-off H1 (100 PFU per cell) in the presence of 10 μg/ml agonists of TLR3 (Poly I:C) or TLR4 (LPS, IMM); 24 h after transfection cells were stained with primary (H1-specific) and secondary fluorochrome labeled antibodies. The mean fluorescence (MFI) of H1-positive DCs in the test samples was detected by flow cytometry. Shown are M ± SD, statistically significant (p < 0.05) differences are indicated by asterisks. Figure S2. Effect of TLR3- and TLR4-activated APCs on the reactivation of CD4+ and CD8+ T-cells. Balb/c mice were immunized (i.m.) with 108 PFU rAdTet-off H1. Forty days after immunization, the pool of CD8+ (a, c) and CD4+ (b, d) T cells from the spleen of euthanized immune mice was re-activated in vitro. Sorted CD8+ and CD4+ T cells were co-cultured with bone marrow derived DCs (c, d) or macrophages (MF) (a, b) preloaded with 20 PFU/cell rAdTet-off H1 in the presence of 0–10 μg/ml agonists of TLR3 (Poly I:C) or TLR4 (LPS, IMM). The number of reactivated IFNγ-producing T-cells were detected by ELISPOT and calculated for 1 million spleen cells. Shown are M ± SD, statistically significant differences (p < 0.05) are indicated by asterisks. (PDF 123 kb) [file 12865_2018_264_MOESM1_ESM.pdf]

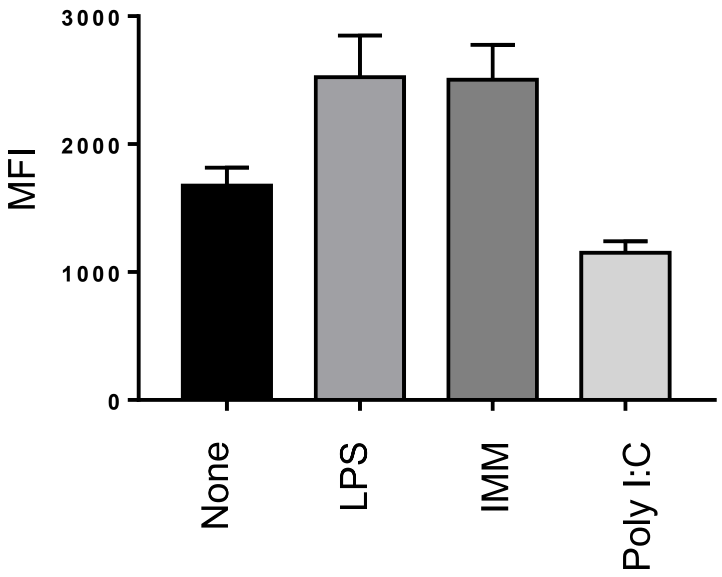

Figure S1. Effect of TLR agonists on the expression of rAd in DCs.

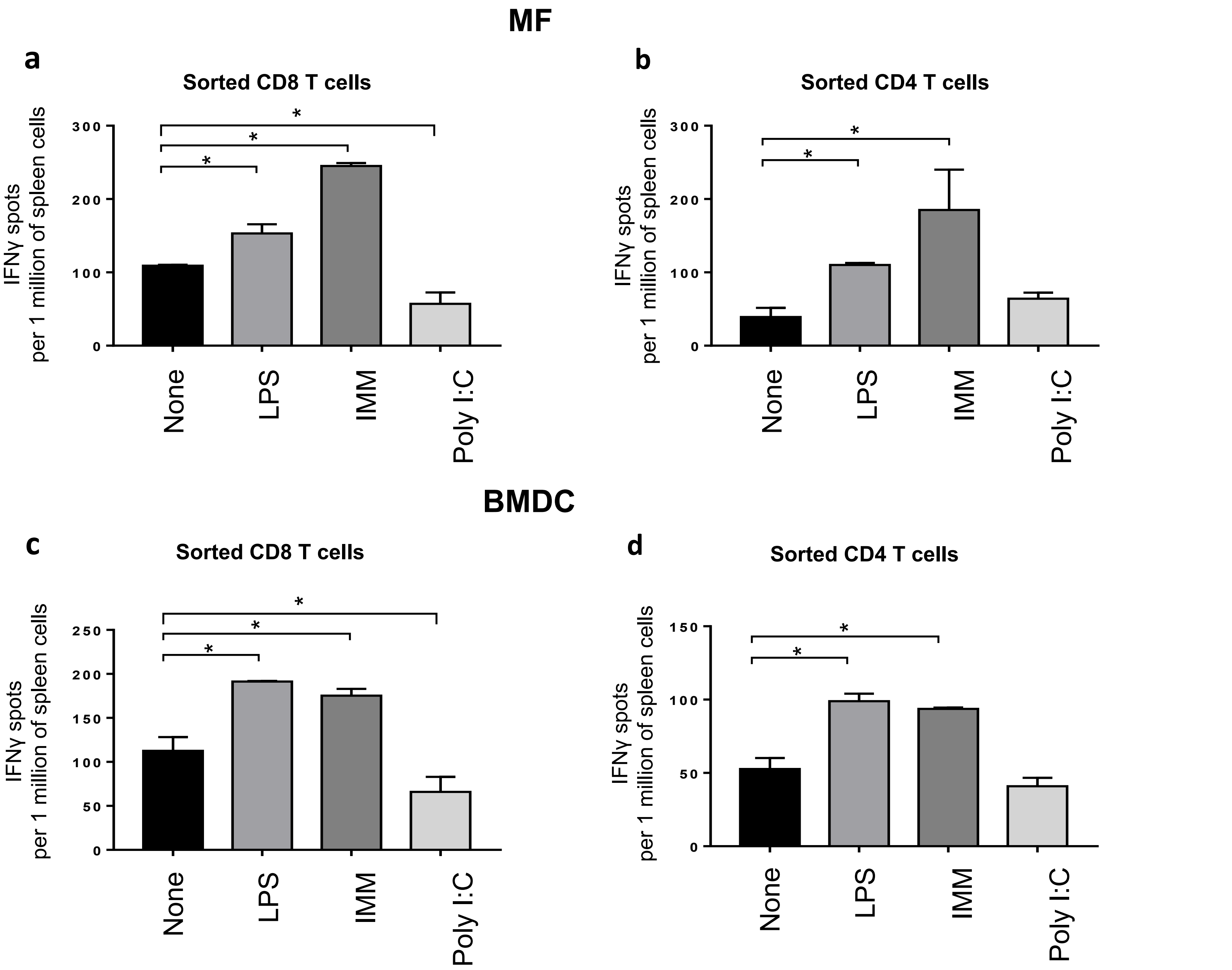

Figure S2. Effect of TLR3- and TLR4-activated APCs on the reactivation of $CD4^+$ and $CD8^+$ T-cells.
